# A Case of Multisystem Inflammatory Syndrome Post-COVID-19 Infection in an Adult

**DOI:** 10.7759/cureus.11961

**Published:** 2020-12-07

**Authors:** Tasnim Ahsan, Bharta Rani

**Affiliations:** 1 Diabetes and Endocrinology, Jinnah Postgraduate Medical Centre, Karachi, PAK; 2 Diabetes & Endocrinology, Orthopaedic & Medical Institute, Karachi, PAK; 3 Diabetes & Endocrinology, Medicell Institute of Diabetes Endocrinology & Metabolism, Karachi, PAK; 4 Diabetes and Endocrinology, Medicell Institute of Diabetes Endocrinology & Metabolism, Karachi, PAK

**Keywords:** multisystem inflammatory syndrome in adult, covid 19

## Abstract

The spectrum of coronavirus disease (COVID-19) continues to evolve as time passes. In the majority of those infected with severe acute respiratory syndrome coronavirus-2 (SARS-CoV-2), apart from fever, respiratory and gastrointestinal symptoms, involvement of other systems, such as cardiovascular and neurological system has also been described. Association between COVID-19 disease and a multisystem inflammatory syndrome in children and adolescents (MIS-C) has now been well defined. However, in adults there are sparse case reports describing a similar phenomenon. This has led to the development of preliminary case definitions for this disease, based on clinical manifestations, laboratory criteria and recent SARS-CoV-2 exposure or infection. Here we present a case of 28-year-old man who presented with high grade fever, rash, gastrointestinal and neurological symptoms fulfilling the criteria of MIS-C with a prior COVID-19 infection and recovered completely in 6 weeks after receiving steroid therapy.

## Introduction

The ongoing pandemic of COVID-19 infection has resulted in an unprecedented health crisis the world over. As we write, the number of positive cases stand at 36,396,118 with a mortality of 1,060,469 worldwide. In Pakistan, the total number of positive patients reported so far is 316,934 and the number of deaths stands at 6,544 (2%) [1]. The disease has a wide spectrum of manifestations with respiratory symptoms being most common. Thus far, there are only a few published case reports on multisystem inflammatory syndrome in adults (MIS-A) [2].

## Case presentation

A 28-year-old married man with thalassemia minor was diagnosed with SARS-CoV-2 infection after experiencing anosmia on 27th May’20, followed by fever for one day with spike of 101^0^F with a positive SARS-CoV-2 RT-PCR done on the same day. Two weeks later, his single RT-PCR for SARS-CoV-2 was negative, and serology was positive. After remaining well for two more weeks, he developed a fever again. He presented to us with a history of high-grade fever with chills spiking up to 105^o^F for 5 days. He was off food, had nausea and vomiting, and was eating very little. He was also complaining of severe aching in legs, generalized weakness, swelling of feet, constipation and some difficulty in voiding urine. There were no respiratory symptoms. For the past two days, he had been taking Ceftriaxone 2g IV OD, with some defervescence, on a presumptive diagnosis of typhoid fever, as malarial parasite and dengue NS1 antigen and antibody were negative.

On examination, he seemed a little withdrawn and restless. He had red eyes bilaterally without any exudation and his face appeared plethoric with a generalized morbilliform rash on the body. Mild edema was noted on the feet. Speech was normal, though he was responding with monosyllables. His BMI was 28.48 kg/m^2^ and vitals were BP 130/70 mmHg, RR 22 breaths/min and HR 110 beats/min. His abdomen was soft and non-tender. There was no motor weakness or sensory loss. Rest of the systemic examination was unremarkable. As he was unable to eat and was vomiting persistently, he was admitted to the hospital. Ceftriaxone was continued during admission. No obvious cause of fever was established. ECG and chest X-ray were normal; ultrasound abdomen reported a fatty liver only. He was discharged home after three days on the family's insistence, after his vomiting and fever had subsided on injectable ondansetron. However, during admission he continued to complain of severe aching in the body, particularly in both legs, and remained a little confused, reluctant to communicate, although obeying all commands. Lumbar puncture was advised but the patient and his family refused.

He came for follow up 6 days later; he was afebrile, complained of difficulty in drinking fluids because of dribbling as he was unable to purse his lips. Additionally, he complained of blurring of vision and intermittent diplopia on lateral gaze. His wife reported a change in behavior and personality, with him having difficulty in processing information. His responses were also inappropriate and not in keeping with his personality. The skin rash had completely subsided and the eyes were minimally congested. He had developed clinical features of bilateral facial nerve palsy. MRI of the brain and orbit was normal. He was seen by an ophthalmologist and was noted to have bilateral optic neuritis and uveitis. The rest of the neurological examination was normal. He was started on oral prednisolone at 1 mg/kg/day, tapering it over 6 weeks. His response was brisk and four weeks into steroid therapy, all his symptoms had abated. After discontinuation of steroids, he remained well.

Results

Complete blood counts showed hypochromic, microcytic anemia (haemoglobin 8.8 g/dl), leukocytosis (13,600 white blood cells/μL) with neutrophilia (10,880 neutrophils/μL) , initially thrombocytopenia (118,000 platelets/μL) later thrombocytosis (686,000 platelets/μL), whereas comprehensive metabolic panel showed normal electrolytes with a sodium of 140 mmol/L, normal hepatic enzymes except raised Gamma GT (rGT 69 U/L) and low albumin (Albumin 2.5 g/dL) and normal muscle enzymes. Notably, his inflammatory markers were raised with an erythrocyte sedimentation rate (ESR) of 58 mmHg, C-reactive protein (CRP) of 13.19 mg/dL (normal range <0.5) and ferritin of 613.9 ng/mL (normal range 20-250); bacterial cultures were negative. SARS-CoV-2 PCR was not done during this admission. Tests for autoimmunity could not be done due to financial constraints.

## Discussion

Post SARS-CoV-2 infection, MIS-C and Kawasaki-like disease has now been well described in children. However, the occurrence of a similar disease has not been seen frequently in adults [3,4]. A few patients with neurological involvement, including Guillain Bare Syndrome, have been described post-COVID-19 infection [5]. In this case, the patient appeared to have wide spread neurological involvement affecting peripheral nerves, intracranial nerves as well as autonomic nervous system. This was heralded by systemic features of acute inflammation with high grade fever, cutaneous exanthem, conjunctivitis and features of peripheral neuropathy. Still later in the course of illness, the cerebral changes and further neurological manifestations emerged with involvement of multiple cranial nerves and worsening encephalopathy. All these features were rapidly alleviated after the institution of steroid therapy. Other than the age limit, our patient fits the MIS-C case definition criteria of both CDC and WHO [6,7]. Although our patient initially presented with thrombocytopenia, over subsequent days, he developed significant thrombocytosis. A case of Kawasaki-like disease in an adult has also been reported with rapid resolution of clinical and laboratory features after using methylprednisolone pulse therapy, IVIG and aspirin with tapering prednisolone thereafter [8]. Another case of MIS post-COVID-19 in an adult has been reported, who met the American Heart Association (AHA) criteria for Kawasaki disease and showed complete resolution with low molecular weight heparin, IVIG and Tocilizumab [9]. Severe SARS-CoV-2 infection also causes hyperinflammation and multiorgan system involvement with respiratory failure as the predominant manifestation. Contrary to that, MIS-A is associated with minimal respiratory symptoms, hypoxemia or radiological abnormalities. In a recently published case series, case definition of MIS-A was described as per following criteria: 1) severe illness requiring hospitalization in a person aged ≥21 years, 2) a positive test result for current or previous SARS-CoV-2 infection during admission or in the previous 12 weeks, 3) severe dysfunction of one or more extrapulmonary organ systems, 4) laboratory evidence of severe inflammation and 5) absence of severe respiratory illness [2]. In our case, the confusion between active COVID-19 disease versus MIS-A did not arise as the patient had tested SARS-CoV-2 positive approximately four weeks prior to presenting to us. This patient had clear documentation of viral clearance from the respiratory tract by virtue of a negative PCR ten days after the positive test and documented seroconversion to SARS-CoV-2 antibodies as per our National Institute of Health guidelines [10]. Persistence of the virus in other organs, such as nervous, cardiovascular, gastrointestinal systems and kidneys, is purported as a possible mechanism for post-COVID MIS-A. Endothelial inflammation and thrombo-inflammatory disease may also contribute to this presentation. In the case of this patient, the predominant involvement was in the nervous, dermatological and gastrointestinal systems; the respiratory system being spared in the initial illness as well as during the diagnosis of MIS-A. This is in keeping with the other described cases [2] and also the brisk and favorable response to treatment with steroids.

## Conclusions

It is possible that cases of MIS-A, like the case reported here, are not being recognized and reported. The initial SARS-CoV-2 infection may go unnoticed either because of the lack of symptoms or facility for testing or false negative results. More research is required to further clarify the risk factors and the mechanisms underpinning MIS-A post-COVID-19 infection.
